# PGC-1α modulates necrosis, inflammatory response, and fibrotic tissue formation in injured skeletal muscle

**DOI:** 10.1186/s13395-016-0110-x

**Published:** 2016-11-08

**Authors:** Ivana Dinulovic, Regula Furrer, Sabrina Di Fulvio, Arnaud Ferry, Markus Beer, Christoph Handschin

**Affiliations:** 1 Biozentrum, University of Basel, Klingelbergstrasse 50/70, CH-4056 Basel, Switzerland; 2 Thérapie des maladies du muscle strié INSERM U974 - CNRS UMR7215 - UPMC UM76, Institut de Myologie and University Rene Descartes, 47, bld de l’Hôpital, G.H. Pitié-Salpétrière, 75013 Paris, France

**Keywords:** PGC-1α, Regeneration, Inflammation, Fibrosis, Macrophages, Necrosis

## Abstract

**Background:**

Skeletal muscle tissue has an enormous regenerative capacity that is instrumental for a successful defense against muscle injury and wasting. The peroxisome proliferator-activated receptor γ coactivator 1α (PGC-1α) exerts therapeutic effects in several muscle pathologies, but its role in damage-induced muscle regeneration is unclear.

**Methods:**

Using muscle-specific gain- and loss-of-function models for PGC-1α in combination with the myotoxic agent cardiotoxin (CTX), we explored the role of this transcriptional coactivator in muscle damage and inflammation.

**Results:**

Interestingly, we observed PGC-1α-dependent effects at the early stages of regeneration, in particular regarding macrophage accumulation and polarization from the pro-inflammatory M1 to the anti-inflammatory M2 type, a faster resolution of necrosis and protection against the development of fibrosis after multiple CTX-induced injuries.

**Conclusions:**

PGC-1α exerts beneficial effects on muscle inflammation that might contribute to the therapeutic effects of elevated muscle PGC-1α in different models of muscle wasting.

**Electronic supplementary material:**

The online version of this article (doi:10.1186/s13395-016-0110-x) contains supplementary material, which is available to authorized users.

## Background

Despite being a mostly postmitotic tissue with very high cellular plasticity, skeletal muscle has a tremendous capacity for regeneration upon injury. The regenerative process in skeletal muscle is highly organized and complex, requiring the coordinated activation of multiple cells types and factors [1]. Heavily damaged muscle fibers undergo necrosis, and as a consequence, muscle-resident mast cells and macrophages are first activated to eradicate cell debris and ultimately make room for newly formed muscle fibers [2]. By secreting cytokines such as tumor necrosis factor α (TNFα) and interleukin 6 (IL-6), these cells subsequently attract neutrophils, which invade the damaged area within hours [2]. Neutrophils in turn contribute to the secretion of a myriad of chemokines including monocyte chemoattractant protein 1 (MCP-1) and macrophage inflammatory proteins (MIP-1α, MIP-1β), which promote the invasion of monocytes from the blood stream.

Resident tissue macrophages and invading monocytes are polarized into the pro-inflammatory M1-type to phagocyte necrotic tissue and promote an environment that favors satellite cell activation and proliferation. Over a period of several days, M1 macrophages are replaced by M2 macrophages, whose role is to suppress the process of inflammation and produce extracellular matrix components that serve as scaffold for the formation of new muscle tissue. The M2 macrophages are thus essential for the switch from the proliferative to the differentiation stage of regeneration. Importantly, timely attraction of macrophages, the switch between subclasses, and the precise termination of the activity of these cells at the end of the regenerative process are prerequisites for the successful activation, proliferation, and differentiation of muscle progenitor cells. For example, despite their anti-inflammatory and pro-regenerative properties, prolonged presence of M2 macrophages leads to uncontrolled activation and proliferation of fibroblasts and overproduction of extracellular components resulting in fibrosis [3]. This excessive formation of fibrous tissue prevents the full functional recovery of muscle tissue.

Exercise training not only boosts the resistance of muscle against damage but might also enhance muscle fiber regeneration by expanding the satellite cell pool amongst other adaptions [4]. In addition, the transition from myoblast proliferation to myofiber differentiation is marked by a prominent switch from glycolysis to oxidative metabolism of glucose and lipids [5]. Intriguingly, the peroxisome proliferator-activated receptor γ coactivator 1α (PGC-1α) is a key regulator of endurance exercise adaptation in skeletal muscle and promotes oxidative metabolism in muscle and other organs [6]. Accordingly, transgenic overexpression of PGC-1α in muscle is sufficient to trigger a shift from glycolytic to oxidative muscle fibers [7] and a high endurance phenotype [8]. Muscle-specific PGC-1α knockout mice inversely exhibit reduced endurance, dysregulation of glucose homeostasis, fiber damage, and systemic inflammation [9]. Due to its potent effect on muscle plasticity, PGC-1α overexpression protects muscles against denervation- and hind limb unloading-induced fiber atrophy [10, 11], sarcopenia [12], Duchenne muscular dystrophy in *mdx* and dystrophin/utrophin double-knockout mice [13–15], a mitochondrial myopathy [16], or muscle wasting promoted by statin drugs [17]. While several candidate mechanisms for this broad therapeutic effect of PGC-1α have been proposed [18], it is still unclear how PGC-1α reduces muscle damage and wasting in these highly divergent disease contexts. Intriguingly, in all of these studies, PGC-1α improved not only muscle function but also muscle cell morphology and integrity, implying a potential role for PGC-1α in fiber repair and regeneration.

Thus, to study the involvement of PGC-1α in muscle damage and inflammation, we assessed the consequence of specific gain- and loss-of-function of muscle PGC-1α in mice treated with the myotoxic agent cardiotoxin (CTX). PGC-1α-dependent differences in macrophage accumulation, initiation of polarization, and removal of necrotic tissue in earlier stages, as well as fibrosis in later stages of regeneration, were noticeable in these models. Nevertheless, late-stage functional recovery was unaffected, even in mice with an ablation of muscle PGC-1α.

## Methods

### Animals

PGC-1α transgenic mice (mTG) express PGC-1α under the control of muscle creatine kinase (MCK) promoter and have been previously described [7]. A muscle-specific PGC-1α knockout model (mKO) was generated by crossing a PGC-1α^flox/flox^ line [19] with a Myf5-cre line (The Jackson Laboratory, stock number 007845). The genotype of the mice was determined by PCR from toe biopsies using specific primers (Additional file 1). Since the Myf5-cre line used to generate the mKO mice is in a mixed strain background in contrast to the clean C57BL/6 J background of the mTG animals [7], we used separate littermate controls for each genotype (referred to as wild type, WT). In this study, male 8–12-week-old mice were used unless stated otherwise. All experimental procedures performed on the mice were approved by the Cantonal and institutional authorities.

### Endurance capacity

Endurance capacity was determined by using a closed treadmill (Columbus Instruments) with a 5° incline. Mice were acclimatized for 2 days. The endurance tests started at 8 m/min for 3 min, with increasing the speed by 2 m/min every 3 min until exhaustion. Data were collected before and during the running protocol and used for calculating maximal oxygen consumption (VO_2max_) and respiratory exchange ratio (RER) based on indirect calorimetry.

### Cardiotoxin injury

In anesthetized mice (O_2_/sevoflurane, 3 % sevoflurane) kept on a warm plate, the lower limbs were shaved and cleaned using 70 % ethanol. Thirty microliters of control vehicle (phosphate buffered saline (PBS)) or 30 μl (3 μg) of CTX (C9759, Sigma) was injected in the belly of the tibialis anterior (TA) muscle using insulin syringes (U-100, 300 μl, 29 G × 1/2 in.). Mice were sacrificed at various time points after the injury and TA muscles collected, transversally cut into two pieces, and frozen for histology or RNA isolation. In the repeated injury model, CTX was intramuscularly injected three times with a 3-week interval between injections. Three weeks after the last injection, the mice were sacrificed and TAs collected.

### Histology

Half of a TA muscle was placed in OCT (Tissue-Tek, Sakura) in plastic molds and frozen in isopentane precooled in liquid nitrogen. Eight-μm-thick sections were cut with a cryostat (Leica CM1950) and stored at −20 °C. Hematoxylin and eosin (Sigma MHS32, HT110232) staining (H&E), nicotinamide adenine dinucleotide (NADH) staining, and Masson’s trichrome (Sigma HT15, HT1079, HT10132) staining were performed on dried sections fixed with paraformaldehyde (PFA) according to the manufacturer’s instructions and mounted with Eukitt mounting medium (O. Kindler). For NADH staining, sections were incubated with NADH (Sigma, N-8129) and nitroblue (Sigma, N-5514) for 30 min at 37 °C and then mounted. Immunostainings were performed on dried fixed sections, blocked with 3 % bovine serum albumin (BSA) in PBS, followed by primary antibody incubation (laminin ab11575 Abcam, DAPI 62248 Thermo Scientific, CD68 MCA1957GA Serotec). After washing with PBS, sections were incubated with secondary antibodies (donkey-anti-rabbit IgG Alexa647 A31573 Life Techologies, goat-anti-rat IgG Alexa488 A11006 Invitrogen) then washed with PBS and mounted with Vectashield (H-1000 Vector).

### Hydroxyproline measurement

After multiple CTX injections, TA muscles were collected and processed for sprectrophotometric measurement of hydroxyproline content as an estimate of amount of fibrotic tissue. Briefly, whole muscles were homogenized, heated in 37 % HCl, and then incubated with chloramine T and Ehrlich’s reagent before measuring absorbance at 560 nm. Measurements were normalized using a standard curve.

### Macrophage isolation and FACS

To isolate macrophages after CTX injections, TA muscles were minced and digested in 2 mg/ml collagenase A solution (Roche Diagnostics), after which liberated cells where filtered and macrophages recovered through density gradient separation in 30 % Percoll solution. For fluorescence-activated cell sorting (FACS), cells were blocked with Fc-receptor blocker (Innovex Biosciences Cat# NB309-5S) and then stained with antibodies against F4/80, CD80 and CD163 (mouse anti-F4/80 APC conjugated: BioLegend Cat#123116, mouse anti-CD80 Pacific-Blue conjugated: BioLegend Cat#104724, rabbit anti-CD163/M130 polyclonal antibody AlexaFluor488 conjugated: Bioss (LucernaChem) Cat#bs-2527R-A488). For each sample, 50,000 events were collected and gated for total macrophages (F4/80^+^), M1 (F4/80^+^CD80^+^CD163^−^) and M2 (F4/80^+^CD80^-^CD163^+^) subpopulations. The data were analyzed using FlowJo v10. To isolate macrophages from uninjured muscles, TA, gastrocnemius, and quadriceps muscles of adult mice at the age of 20–26 weeks were pooled to obtain enough cells. The procedure was similar as described above, using collagenase B solution (Roche Diagnostics) to digest the muscles. Cell viability was verified by Propidium Iodide (BioLegend Cat#421301).

### Image acquisition and quantification

Images of immunostained muscle sections were acquired with a Zeiss LSM700 microscope using the Zen 2010 software and a 25x objective with 0.5 zoom. H&E, NADH, and Masson’s trichrome staining images were captured using an Olympus IX81 with a 4x objective. The entire area of the muscle sections was acquired and quantification was performed on the complete area using the ImageJ software. Intensity of NADH staining, area of macrophage staining (CD68^+^ area), and necrotic area were measured using the same software. Necrotic tissue assessment was based on the morphology and coloration of the fibers in H&E sections (swollen pink fibers as necrotic vs. small purple fibers as regenerating) as well as with the help of the CD68^+^ cells indicating the actively regenerating area. Fiji together with the Weka plugin was applied for measuring fibrosis. The obtained probability images were subsequently used for assessing fibrotic area using the same threshold for all the samples within one experiment. Quantification was performed in a blinded manner.

### RNA extraction and relative qPCR

Total RNA was extracted from half of a TA muscle using TRI Reagent (Sigma) and lysing matrix tubes (MP Biomedicals) according to the manufacturer’s instructions. After measuring RNA concentration on a Nanodrop 1000 (Thermo Scientific), 1 μg was treated with DNase I (Invitrogen) and used for cDNA synthesis by the reverse transcriptase Superscript II (Invitrogen). Relative messenger RNA (mRNA) levels were measured by quantitative PCR (qPCR) on a StepOne machine with SYBR green-based detection and normalized to TATA binding protein (TBP) expression using the ΔΔCt method. A list of primers is provided in Additional file 1.

### Muscle contractility measurements in situ

TA muscle regeneration was evaluated by measuring in situ isometric muscle contraction in response to nerve stimulation as previously described [20]. Briefly, mice were anesthetized using a pentobarbital solution (ip, 60 mg/kg), and supplemental doses were given as required to maintain deep anesthesia during the experiments. Each hind paw was fixed with clamps to a platform, and the knees were immobilized using stainless steel pins. The distal tendons of the muscles were attached to an isometric transducer (Harvard Bioscience) using a silk ligature. The sciatic nerves were proximally crushed and distally stimulated with a bipolar silver electrode using supramaximal square wave pulses of 0.1 ms duration. All data provided by the isometric transducer were recorded and analyzed using the PowerLab software (4SP, AD Instruments). Isometric measurements were made at an initial length L0 (the length at which maximal tension was obtained during the tetanus). Responses to tetanic stimulation (pulse frequency from 6.25, 12.5, 25, 50, 100, and 143 Hz) were successively recorded and maximal tetanic force (P0) was determined. Muscle mass was measured to calculate specific force (P0 [g]/weight [g]). Finally, fatigue resistance was assessed with one continuous contraction (50 Hz for 45 s), measuring the time to reach 50 % of the initial force. After contractile measurements, the mice were sacrificed with an overdose of anesthetic solution.

### Statistical analysis

All data are presented as individual values or average ± SEM. CTX-injured muscle gene expression values were normalized to the PBS values of WT animals unless stated otherwise. Statistical analysis was performed using Student’s *t* test for comparison of two groups, and *p* ≤ 0.05 was considered significant.

## Results

### Macrophage polarization shift in mTGs

**Fig. 1:**
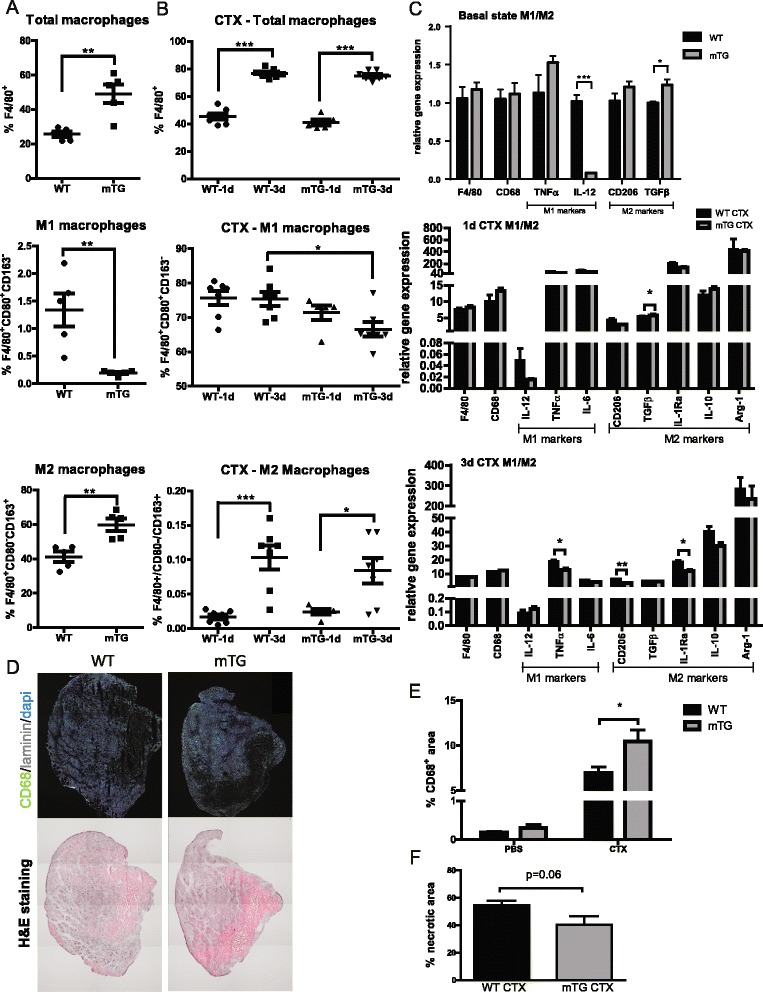
Macrophages and tissue necrosis prior to and in early days after cardiotoxin injury in mTG mice. **a** FACS analysis of uninjured muscles of WT and mTG animals, showing the proportion of pan-macrophages (F4/80^+^ cells), M1 macrophages (F4/80^+^CD80^+^CD163^-^ cells), and M2 macrophages (F4/80^+^CD80^−^CD163^+^ cells); *n* = 5 per group; **b** proportion of pan-macrophages and M1 and M2 subpopulations in TA muscles of WT and mTG animals 1 and 3 days after CTX injection using FACS analysis; *n* = 5–7 per group; **c** relative gene expression levels of M1 and M2 macrophage markers in baseline condition (*n* = 5–6 per group) as well as 1 day (*n* = 3–4 per group) and 3 days (*n* = 5–7 per group) post-CTX-injection; **d** representative immunohistochemistry (IHC) images (CD68: *green*; dapi/nuclei: *blue*; laminin: *gray*) and corresponding H&E sections showing accumulation of macrophages (*green* in IHC images) in actively regenerating areas (*dark purple* in H&E staining); **e** measurement of area occupied by M1 macrophages from IHC images; *n* = 7–8 per group; **f** measurement of necrotic area from H&E sections normalized to total section area; *n* = 7–8 per group (*t* test); values are plotted as individual values or average ± SEM; *p ≤ 0.05, **p ≤ 0.01, ***p ≤ 0.001

Activation of macrophages and the subsequent shift from the M1- to the M2-type are physiological processes which unfold during regeneration and are associated with the progression from myoblast proliferation towards their fusion [3]. In order to assess the baseline condition regarding abundance of pan- as well as polarized M1 and M2 macrophages, we performed FACS analysis of muscles of untreated mice. In this context, mTG mice have approximately 25 % more F4/80^+^ macrophages compared to WT animals (Fig. 1a). Although there is a significant difference between WT and mTGs regarding the proportion of F4/80^+^CD80^+^CD163^-^ M1 macrophages, this difference is very small (1.3 % in WT vs 0.2 % in mTG) and the amount of M1 macrophages seems neglectable in healthy muscles. In contrast, a large proportion of the tissue resident macrophages are polarized towards an M2 phenotype (~40 % in WT and ~60 % in mTG). The higher proportion of polarized M2 macrophages in mTG animals suggests that the muscles of these animals might be preconditioned for faster repair and regeneration. This preconditioning is also partly supported by qPCR data showing higher expression levels of TGFβ and a substantial reduction of IL-12 expression in basal, non-injured state (Fig. 1c). To investigate whether the preconditioning of these mice also alter the regeneration kinetics, CTX was injected into the TA muscles and abundance of pan-macrophages (F4/80^+^) as well as the relative numbers of M1 (F4/80^+^CD80^+^CD163^-^) and M2 (F4/80^+^CD80^-^CD163^+^) macrophages were measured by FACS. At 1 and 3 days post-injection, a remarkable infiltration of macrophages was observed (Fig. 1b) with no difference between WT and mTG animals. One day after the injection, the proportion of M1 macrophages was ~75 % whereas the amount of M2 macrophages was very low in both genotypes. In mTG animals, the proportion of M1 macrophages dropped to 66 % at day 3 and was approximately 10 % lower compared to WT animals.

One of the main roles of M1 macrophages is the removal of necrotic debris, which could be impaired in the mTG mice due to the faster decrease in the proportion of M1 macrophages. We therefore performed H&E stainings in order to assess the overall morphology of the muscle tissue 4 days after CTX, combined with immunohistochemical stainings for CD68^+^ macrophage-covered area on consecutive sections (Fig. 1d). Interestingly, by measuring the area occupied by swollen purple fibers on H&E sections, we detected a trend towards smaller necrotic areas in mTG mice compared to WT controls (Fig. 1f). Complementary to those, the areas of macrophage-positive staining on immunohistological sections that correspond to actively regenerating areas on H&E sections were significantly larger in injured mTGs (Fig. 1e). This suggests that activation of macrophages is enhanced in mTG animals and may contribute to a faster regeneration. Alternatively, a potentially protective environment in the uninjured mTG muscle could reduce tissue necrosis and therefore require less M1 activation. In either case, the earlier drop in M1 macrophage number in the mTG could thus be a consequence of an accelerated clean-up of necrotic tissue.

### Multiple CTX injury results in reduced fibrosis in mTG mice

**Fig. 2:**
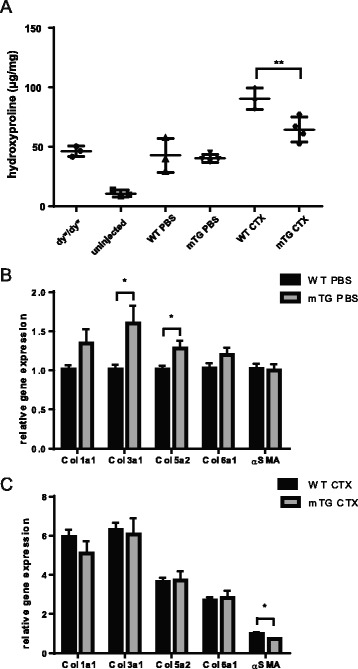
Fibrosis after multiple cardiotoxin injections in mTG mice. **a** Hydroxyproline measurement in mTG mice 3 weeks after the third CTX injection; *n* = 3–4 per group; muscles from laminin α2-deficient (dy^w^) and uninjected WT mice served as positive and negative controls, respectively; **b**, **c** Relative mRNA levels of pro-fibrotic genes at same the time point in PBS-injected muscles (**b**) and CTX-injected muscles (**c**); CTX-injured muscle gene expression levels were normalized to PBS levels of WT animals; *n* = 7–10 per group (*t* test); values are plotted as individual values or average ± SEM; *p ≤ 0.05, **p ≤ 0.01; Abbreviations: *Col* collagen

The modulation of macrophage polarization observed in mTGs could have other negative consequences. For example, prolonged activation of macrophages can lead to expansion of fibroblasts and excessive secretion of extracellular matrix (ECM) components [21]. Although these factors contribute to the regenerative process, their removal at later stages of regeneration is essential in order to fully restore functional skeletal muscle tissue and to avoid excessive fibrotic tissue formation. Therefore, to study fibrosis, repeated CTX injuries as a model of chronic damage were used in order to exacerbate the degeneration/regeneration process and increase fibrotic tissue formation. Three weeks after the last of the three CTX injections, we measured the hydroxyproline content in the whole muscle lysate as an indicator of fibrosis. Intriguingly, decreased fibrosis in the mTGs was detected (Fig. 2a), in line with a higher drop in expression of several collagens and α-smooth muscle actin (α-SMA) in the transgenic model between PBS- and CTX-injected muscles (Fig. 2b, c).

### Increased fibrosis and reduced necrotic debris clearance in mKO mice

The modulation of macrophage polarization and the inhibition of fibrosis in the repeated injury model imply a beneficial effect of muscle PGC-1α on regeneration in the mTG model. We therefore wanted to assess whether muscle PGC-1α was required for proper clearance of necrosis and fibrosis in mice with a muscle-specific ablation of PGC-1α (mKOs).

In order to study the role of PGC-1α in early events of skeletal muscle regeneration, we generated muscle-specific knockout mice (mKO) using myogenic factor 5 (Myf5)-cre to delete PGC-1α already at the muscle progenitor stage. Analysis of these Myf5-mKO mice confirmed the specific reduction in PGC-1α transcript levels in different muscle beds as well as brown adipose tissue (BAT) (Additional file 2A). Similar to other reported PGC-1α mKO lines [9], endurance exercise capacity and maximal oxygen consumption were reduced in Myf5-mKOs (Additional file 2B–D). In contrast, the respiratory exchange ratio (RER) at rest or during exercise trials was unchanged between the genotypes (Additional file 2E). Accordingly, the expression of several genes encoding mitochondrial regulatory proteins was reduced (Additional file 3B).

**Fig. 3:**
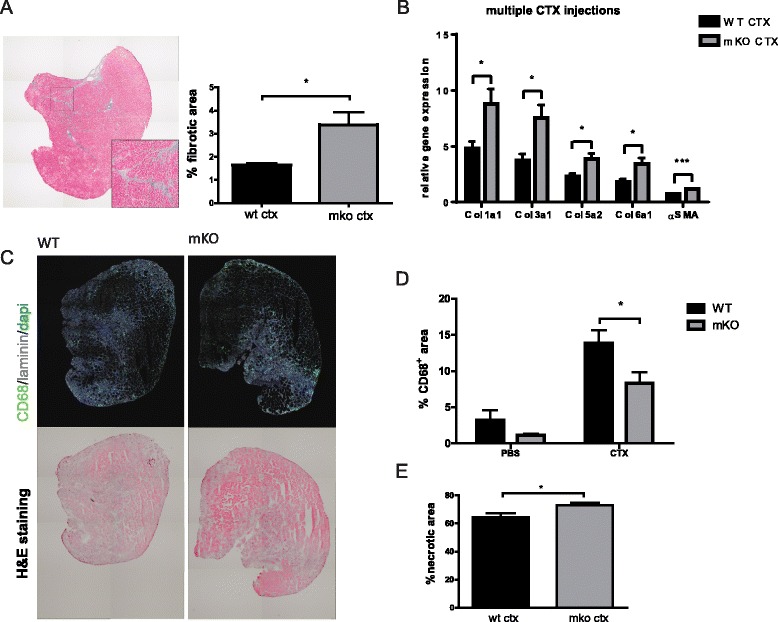
Fibrosis, necrosis, and M1 macrophages after cardiotoxin injury in mKO mice. **a** Representative Masson’s trichrome stained section with enlarged area and quantification of fibrotic area in mKO mice 3 weeks after the third CTX injection; *n* = 4–7 per group; **b** relative mRNA levels of pro-fibrotic genes at the same time point; CTX-injured muscle gene expression levels were normalized to PBS levels of WT animals; *n* = 6–7 per group (*t* test); **c** representative IHC images (CD68: *green*; dapi/nuclei: *blue*; laminin: *gray*) and corresponding H&E sections showing accumulation of macrophages (*green* in IHC images) in actively regenerating area (*dark purple* in H&E staining); **d** measurement of area occupied by M1 macrophages from IHC images; *n* = 5 per group; **e** measurement of necrotic area from H&E sections normalized to total section area; *n* = 5 per group; values are plotted as average ± SEM; *p ≤ 0.05, ***p ≤ 0.001

Indeed, increased fibrotic area (Masson’s trichrome staining) and higher expression of collagens and α-SMA were detected in the mKOs compared to WT littermates after multiple CTX injections (Fig. 3a, b). Moreover, at 4 days after CTX, reduced clearance of necrotic debris together with a tendency to smaller regenerating CD68^+^ macrophage area was observed in the loss-of-function model for muscle PGC-1α (Fig. 3c–e). Interestingly, the elevation of the expression of the M1 markers TNFα and IL6 at 4 days post-CTX (Additional file 4) indicated sustained activation of inflammation compared to the mTG data (Fig. 1c). The results therefore imply a slower inflammatory response to injury in muscle PGC-1α loss-of-function.

### Functional recovery of CTX-injected TA in mTG and mKO mice at 10 days after injury

**Fig. 4:**
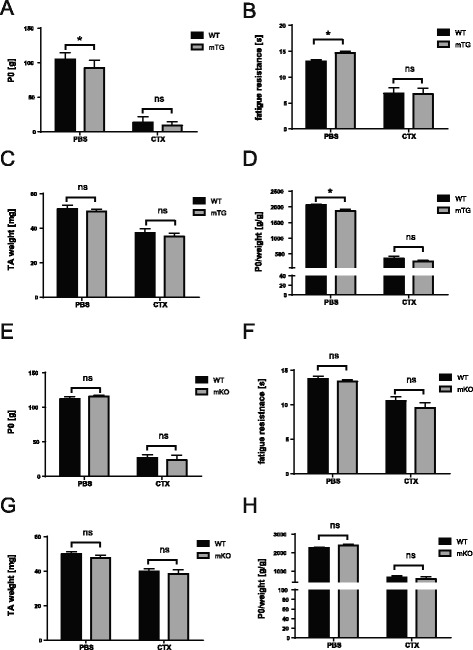
*In situ* TA contractility measurements 10 days after cardiotoxin injury in mTG and mKO mice. Absolute maximal force (P0) in **a** mTG and **e** mKO mice; fatigue resistance in **b** mTG and **f** mKO mice; TA weight in **c** mTG and **g** mKO mice; specific maximal force (P0/weight) in **d** mTG and **h** mKO mice; *n* = 8 per group for mTGs and *n* = 7–11 per group for mKOs; values are plotted as average ± SEM; *p ≤ 0.05

Based on the modulation of necrosis and fibrosis by muscle PGC-1α, we subsequently studied functional late-stage regeneration after CTX-induced muscle injury using in situ contractility measurements on control (PBS-injected) and injured (CTX-injected) TA muscles. At the time point measured (10 days after CTX injection), muscle functionality was partially restored. Surprisingly, no difference in fatigue resistance or absolute maximal force was found in any of the genotypes (Fig. 4). Thus, specific gain- and loss-of-function of muscle PGC-1α affect early events in repair and regeneration without altering the later stage of functional recovery.

## Discussion

Proper muscle regeneration is not only important in the context of pathological muscle injuries but also essential for the incremental adaptation of muscle to exercise [22]. Training not only offers protection against further insults by altering muscle-intrinsic functions including elevated oxidative capacity [23–25] but also results in an acceleration of the regeneration process through increased vascularity [26], adaptations of the neuromuscular junction [27], and other muscle extrinsic changes [28]. Muscle PGC-1α is a key regulator of endurance training adaptation and confers beneficial effects on muscle fiber integrity and muscle functionality in a variety of different muscle diseases. Using skeletal muscle-specific gain- and loss-of-function animal models for PGC-1α, we now describe how PGC-1α affects macrophage-mediated clearance of necrotic tissue and formation of fibrotic tissue in the context of CTX-induced muscle damage.

Similar to the exercise-induced preconditioning of muscles to improve regeneration, muscle-specific overexpression of PGC-1α seems to be sufficient to prime the muscle for faster regeneration, implied by the higher number of pan- and a higher proportion of M2 macrophages. Modulation of necrosis clearance by altered levels of PGC-1α correlated with a modulation of tissue macrophage number and/or activity as well as the area occupied by CD68^+^ macrophages. Intriguingly, since the overall macrophage infiltration following CTX injection remains unchanged between the WTs and mTGs, the drop of M1-type macrophages after 3 days implies a faster clean-up of necrosis and ensuing deactivation of M1 macrophages in animals with elevated muscle PGC-1α. Hence, although our experimental design was too short to visualize the expected switch from M1 to M2 macrophages, the mTG mice have a potentially accelerated early regeneration based on the lower proportion of M1 macrophages. In fact, the PGC-1α-dependent acceleration of initiation of clearance of necrosis correlates with a protection against fibrosis in animals with a repetitive CTX-induced muscle injury. Inversely, the possible delay not only in initiation but also in termination of different regenerative events, as for example manifested by the delayed clearance of necrotic areas, could contribute to the increased fibrosis after multiple CTX injuries in mKO animals.

Importantly, our two mouse models are not mirrored in terms of the timing of overexpression and knockout of PGC-1α. The expression of the transgene under the MCK promoter in mTGs is expected in the adult myofiber. In mKO mice, gene ablation is initiated already at the muscle precursor stage driven by cre expression controlled by the Myf5 promoter. As a consequence, the observed changes in the early stages of regeneration are almost certainly indirectly affected by the transgene, whereas in the mKO model, both direct and indirect effects are conceivable. Thus, to exclude possible confounding factors related to the contribution of satellite cells to regeneration in mKOs, we measured the kinetics of myogenic regulatory factor (MRF) gene expression before as well as at several time points after CTX injury (Additional file 5A–E). Although the kinetics seems slightly slower in the mKO model, no difference was observed in the recovery of the functionality (Fig. 4e–h) of the tissue.

Muscle PGC-1α expression has previously been associated with tissue inflammation. For example, a persistent low-grade fiber damage in mKO animals is linked to elevated pro-inflammatory gene expression in muscle as well as systemic elevation of IL-6 and TNFα that might lead to detrimental effects in other organs, e.g., pancreatic β-cells [29]. In line, reduced muscle PGC-1α expression in glucose intolerant and type 2 diabetic patients significantly correlates with elevated IL-6 and TNFα [29]. Inversely, elevation of PGC-1α in muscle cells results in decreased activity of the nuclear factor κB (NF-κB), the main regulator of pro-inflammatory gene expression [30]. Importantly however, in other contexts, the intrinsic effects of PGC-1α on muscle inflammation is most likely masked by the much higher levels of these genes in resident and infiltrating immune cells. For example, in mice exposed to strong pro-inflammatory stimuli, PGC-1α modulation in skeletal muscle affects the expression of pro- and anti-inflammatory genes [31, 32]. Similarly, the massive muscle damage elicited by CTX injection leads to an elevation of immune cell activation as reported here. Thus, the interplay between muscle PGC-1α, inflammation, and immune cells is multi-faceted and highly context-dependent [33, 34].

The changes in inflammatory and fibrotic processes described in mTG mice are probably an indirect effect of muscle PGC-1α-dependent remodeling of non-injured tissue since muscle PGC-1α levels drop rapidly after CTX-induced damage in WT and in mTG mice (Additional file 6A) and slowly return to pre-injury levels in both genotypes (Additional file 6B–E). Concomitantly, a sharp reduction in PGC-1α-regulated mitochondrial genes and recovery of their expression and functionality was detected after injury, following the expression pattern of PGC-1α in this context (Additional file 3). Based on these observations, we hypothesize that PGC-1α overexpression preconditions the intact muscle for a swift response to injury. For example, the elevated levels of M2 macrophages, the substantial reduction of the pro-inflammatory IL-12, and the elevation of the anti-inflammatory TGFβ could point towards a more protective environment in the muscles of mTG mice. It is conceivable that subsequent to damage, the higher abundance of macrophages and altered expression of various chemo- and cytokines help to initiate a chain of events that collectively accelerate multiple steps in early muscle regeneration, e.g., upon CTX injury in mTGs. The secreted phosphoprotein 1 (Spp1) is an example for a PGC-1α-regulated factor produced and secreted from muscle cells that activates macrophages even in non-injured skeletal muscle tissue [35]. Furthermore, many of the observed effects could at least in part be attributed to differential expression of myostatin (Mstn) in both of our mouse models prior to injury (Additional file 6F, G). Mstn is a member of TGFβ family and a known inducer of fibrosis [36]. Accordingly, Mstn^−/−^ mice are protected against fibrosis formation upon notexin injury [37]. At the same time, Mstn^−/−^ mice exhibit faster recruitment of macrophages, comparable to mTG mice. Therefore, Mstn^−/−^ and PGC-1α overexpression mouse model share similar regenerative phenotypes, and consequently, the reduction of Mstn levels in mTGs that exists already prior to injury could contribute to the differences observed in the muscle-specific PGC-1α gain-of-function mouse model. Mstn expression drops after injury in WT animals and slowly returns to pre-injury levels [38] (Additional file 6F, G). In addition, lower and higher levels of Mstn were detected late in the regeneration process in mTG and mKO animals, respectively (Additional file 6F, G). The contribution of Mstn to the modulation of muscle regeneration downstream of PGC-1α will have to be tested: such a specific involvement however could provide an explanation for the regulation of Mstn by PGC-1α in the absence of alteration of muscle mass [6].

## Conclusions

In summary, we now provide evidence that muscles with higher levels of PGC-1α are preconditioned for faster resolution of inflammation and necrosis as well as a prevention of fibrosis after repeated injury. Besides its central role in exercise adaptation, the effect of PGC-1α on regeneration might contribute to the therapeutic potential of elevation of PGC-1α in different muscle diseases and thus reveal novel potential approaches for the treatment of such pathologies.

## Additional files

Additional file 1: List of qPCR primers. (DOC 47 kb)
Additional file 2: Characterization of Myf5-mKO mice. A) Relative PGC-1α mRNA expression levels in various organs; mRNA levels in mKO mice were normalized to WT (control littermate) levels; *n* = 6 per group; exercise capacity in mKO mice is reduced: B) time until exhaustion, C) maximal speed reached, D) VO_2_ max, and E) respiratory exchange ratio (RER) values before run and at exhaustion; *n* = 8–9 per group; values are plotted as average ± SEM; **p* ≤ 0.05, ***p* ≤ 0.01, ****p* ≤ 0.001; Abbreviations: Gastro (gastrocnemius), Sol (soleus), QU (quadriceps), TA (tibialis anterior), Edl (extensor digitorum longus), Diaph (diaphragm), BAT (brown adipose tissue), WAT sc (white adipose tissue subcutaneous), WAT vis (WAT visceral). (PDF 552 kb)
Additional file 3: PGC-1α functionality before and after cardiotoxin injection. A-B) Relative mRNA expression levels of mitochondrial regulatory genes in PBS-injected muscles of mTGs (*n* = 7–8 per group) (A) and mKO mice (*n* = 5 per group) (B) 4 days after injection; (C–D) Relative mRNA expression levels in CTX-injected muscles of mTGs (*n* = 7–8 per group) (C) and mKO mice (*n* = 5 per group) (D) 4 days after injection; E-F) NADH staining of TA 19 days after PBS or CTX injections and signal intensity measurement in mTG (*n* = 8–10 per group) (E) and mKO (*n* = 5 per group) mice (F); (t-test). Lower values indicate darker staining (signal intensity 0–255, where 0 corresponds to fully saturated signal and 255 to no signal); values are plotted as average ± SEM; **p* ≤ 0.05, ***p* ≤ 0.01, ****p* ≤ 0.001; Abbreviations: PRC (PGC-1-related coactivator), Tfam (mitochondrial transcription factor A), Gabpa (GA-binding protein alpha). (PDF 1565 kb)
Additional file 4: M1 and M2 marker expression in mKO mice before and in early days after cardiotoxin injury. Relative gene expression A) in the basal state in mKO mice (*n* = 4–6 per group), B) 2 days (*n* = 4 per group), and C) 4 days (*n* = 5 per group) after CTX in mKO mice; CTX-injured muscle gene expression levels were normalized to PBS levels of WT animals; values are plotted as average ± SEM; **p* ≤ 0.05, ***p* ≤ 0.01. (PDF 525 kb)
Additional file 5: Regeneration in mKO mice. A) Pax7, B) MyoD, C) Myog, and D) MRF-4 gene expression 2 days (*n* = 4 per group), 4 days (*n* = 5 per group), 7 days (*n* = 5 per group), 10 days (*n* = 8–12 per group), and 19 days (*n* = 5 per group) after CTX injury; CTX-injured muscle gene expression levels were normalized to PBS levels of corresponding genotype; E) relative gene expression of the same genes in the basal, uninjured state (*n* = 6 per group); mKO expression was normalized to WT levels (*t* test); values are plotted as average ± SEM; **p* ≤ 0.05, ***p* ≤ 0.01. (PDF 535 kb)
Additional file 6: PGC-1α and Mstn gene expression during course of cardiotoxin-induced regeneration. Relative mRNA levels of PGC-1α in mTG mice A) 8 h (*n* = 4–6 per group), B) 4 days (7–8 per group), C) 7 days (*n* = 5 per group), D) 10 days (*n* = 8 per group), and E) 19 days (*n* = 8–10 per group) post-CTX; relative mRNA levels of Mstn 19 days after single CTX injection in F) mTG (*n* = 8–10 per group) and G) mKO (*n* = 5 per group) mice; values are plotted as average ± SEM; **p* ≤ 0.05, ****p* ≤ 0.001. (PDF 185 kb)

## Abbreviations

CTX: Cardiotoxin
mKO: Muscle-specific PGC-1α knockout mice
mTG: Muscle-specific PGC-1α transgenic mice
PGC-1α: Peroxisome proliferator-activated receptor γ coactivator 1α
TA: Tibialis anterior
