# Supplementary material for: PGC-1α modulates necrosis, inflammatory response, and fibrotic tissue formation in injured skeletal muscle
Source: Skelet Muscle. 2016 Nov 8;6:38. doi: 10.1186/s13395-016-0110-x (PMC5101792; doi:10.1186/s13395-016-0110-x)
Supplement: Additional file 1: — List of qPCR primers. (DOC 47 kb) [file 13395_2016_110_MOESM1_ESM.doc]

Additional File 1. List of qPCR primers

| **Target** | **Fwd primer (5' - 3')** | **Rev primer (5' - 3')** |
| --- | --- | --- |
| Arg-1 | TGGCTTGCGAGACGTAGAC | GCTCAGGTGAATCGGCCTTTT |
| α-SMA | GTC CCA GAC ATC AGG GAG TAA | TCG GAT ACT TCA GCG TCA GGA |
| CD206 | GTGGATTGTCTTGTGGAGCA | TTGTGGTGAGCTGAAAGGTG |
| CD68 | CCAATTCAGGGTGGAAGAAA | CTCGGGCTCTGATGTAGGTC |
| Col1a1 | GCT CCT CTT AGG GGC CAC T | CCA CGT CTC ACC ATT GGG G |
| Col3a1 | CTG TAA CAT GGA AAC TGG GGA AA | CCA TAG CTG AAC TGA AAA CCA CC |
| Col5a2 | TTG GAA ACC TTC TCC ATG TCA GA | TCC CCA GTG GGT GTT ATA GGA |
| Col6a1 | CTG CTG CTA CAA GCC TGC T | CCC CAT AAG GTT TCA GCC TCA |
| ERRα | GCA GGG CAG TGG GAA GCT A | CCT CTT GAA GAA GGC TTT GCA |
| F4/80 | CTTTGGCTATGGGCTTCCAGTC | GCAAGGAGGACAGAGTTTATCGTG |
| Gabpa | AGC GCA TCT CGT TGA AGA AG | TCC TGC TCT TTT CTG TAG CCT |
| IL-10 | CTGGACAACATACTGCTAACCG | GGGCATCACTTCTACCAGGTAA |
| IL-12 | GCT TCT CCC ACA GGA GGT TT | CTA GAC AAG GGC ATG CTG GT |
| IL-1Ra | AAATCTGCTGGGGACCCTAC | TGAGCTGGTTGTTTCTCAGG |
| IL-6 | CCT TCC TAC CCC AAT TTC CAA | TCC TTA GCC ACT CCT TCT GTG ACT |
| MRF-4 | CGC GAA AGG AGG AGA CTA AAG A | CCA CAG TCC GAC GCT TCA G |
| Mstn | GCT GGC CCA GTG GAT CTA AA | CAG CCC CTC TTT TTC CAC ATT |
| Myf5 | CAT GTG GGC CTG CAA AGC | TGC GCC GAT CCA TGG TA |
| MyoD1 | GCC GGT GTG CAT TCC AA | CAC TCC GGA ACC CCA ACA G |
| Myog | GCA GCG CCA TCC AGT ACA TT | ATC GCG CTC CTC CTG GTT |
| Pax7 | AAA AAA CCC TTT CCC TTC CTA CA | AGC ATG GGT AGA TGG CAC ACT |
| PGC-1α ex2 | TGA TGT GAA TGA CTT GGA TAC AGA CA | CGT CAT TGT TGT ACT GGT TGG ATA TG |
| PGC-1α ex3-5 | AGC CGT GAC CAC TGA CAA CGA G | GCT GCA TGG TTC TGA GTG CTA AG |
| PGC-1β | CCA TGC TGT TGA TGT TCC AC | GAC GAC TGA CAG CAC TTG GA |
| PRC | CAC CCT GCC GGA GTG AAA T | CGC ATT GAC TGC TGC TTG TC |
| TBP | TGC TGT TGG TGA TTG TTG GT | CTG GCT TGT GTG GGA AAG AT |
| Tfam | GGA ATG TGG AGC GTG CTA AAA | TGC TGG AAA AAC ACT TCG GAA TA |
| TGFβ | GAA ACG GAA GCG CAT CGA | TGG CGA GCC TTA GTT TGG A |
| TNF-α | CACAAGATGCTGGGACAGTGA | TCCTTGATGGTGGTGCATGA |
|  |  |  |
| Cre (genotyping) | GCGGTCTGGCAGTAAAAACTATC | GTGAAACAGCATTGCTGTCACTT |
| Lox P (genotyping) | TCC AGT AGG CAG AGA TTT ATG AC | TGT CTG GTT TGA CAA TCT GCT AGG TC |
