# Supplementary figures and images for: PGC-1α modulates necrosis, inflammatory response, and fibrotic tissue formation in injured skeletal muscle
Source: Skelet Muscle. 2016 Nov 8;6:38. doi: 10.1186/s13395-016-0110-x (PMC5101792; doi:10.1186/s13395-016-0110-x)

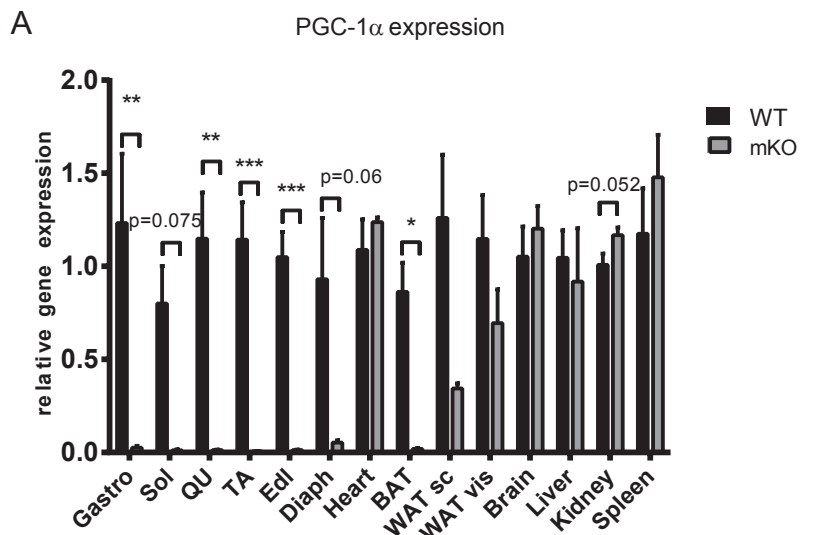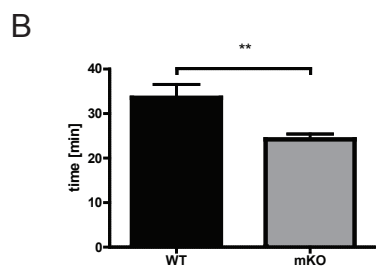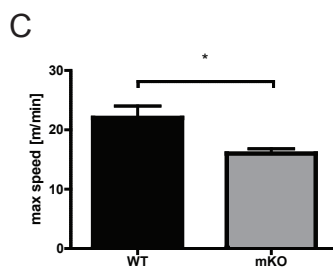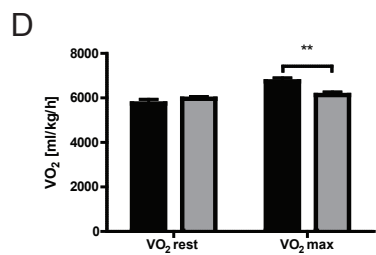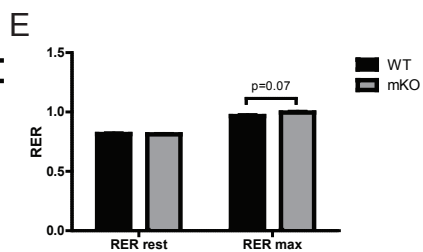

Supplement: Additional file 2: — Characterization of Myf5-mKO mice. A) Relative PGC-1α mRNA expression levels in various organs; mRNA levels in mKO mice were normalized to WT (control littermate) levels; n = 6 per group; exercise capacity in mKO mice is reduced: B) time until exhaustion, C) maximal speed reached, D) VO2 max, and E) respiratory exchange ratio (RER) values before run and at exhaustion; n = 8–9 per group; values are plotted as average ± SEM; *p ≤ 0.05, **p ≤ 0.01, ***p ≤ 0.001; Abbreviations: Gastro (gastrocnemius), Sol (soleus), QU (quadriceps), TA (tibialis anterior), Edl (extensor digitorum longus), Diaph (diaphragm), BAT (brown adipose tissue), WAT sc (white adipose tissue subcutaneous), WAT vis (WAT visceral). (PDF 552 kb) [file 13395_2016_110_MOESM2_ESM.pdf]

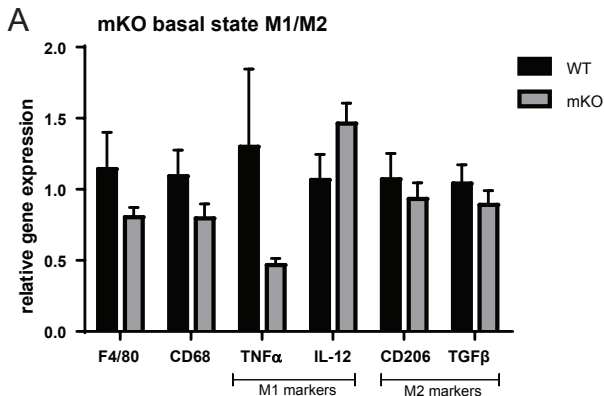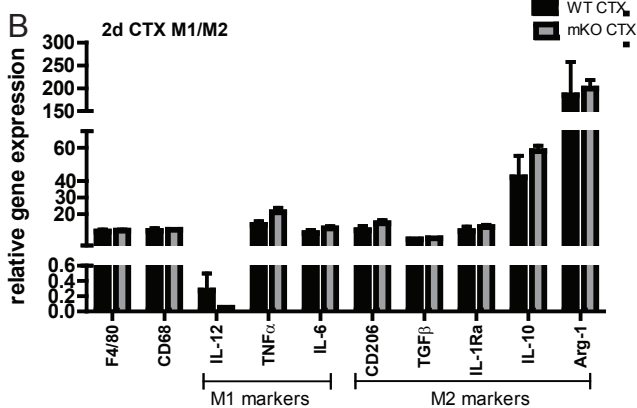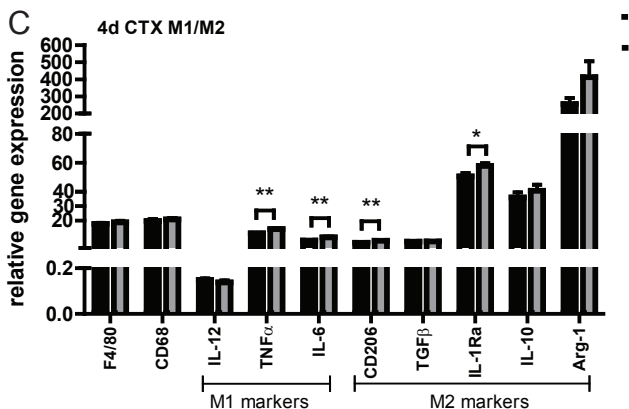

Supplement: Additional file 3: — PGC-1α functionality before and after cardiotoxin injection. A-B) Relative mRNA expression levels of mitochondrial regulatory genes in PBS-injected muscles of mTGs (n = 7–8 per group) (A) and mKO mice (n = 5 per group) (B) 4 days after injection; (C–D) Relative mRNA expression levels in CTX-injected muscles of mTGs (n = 7–8 per group) (C) and mKO mice (n = 5 per group) (D) 4 days after injection; E-F) NADH staining of TA 19 days after PBS or CTX injections and signal intensity measurement in mTG (n = 8–10 per group) (E) and mKO (n = 5 per group) mice (F); (t-test). Lower values indicate darker staining (signal intensity 0–255, where 0 corresponds to fully saturated signal and 255 to no signal); values are plotted as average ± SEM; *p ≤ 0.05, **p ≤ 0.01, ***p ≤ 0.001; Abbreviations: PRC (PGC-1-related coactivator), Tfam (mitochondrial transcription factor A), Gabpa (GA-binding protein alpha). (PDF 1565 kb) [file 13395_2016_110_MOESM3_ESM.pdf]

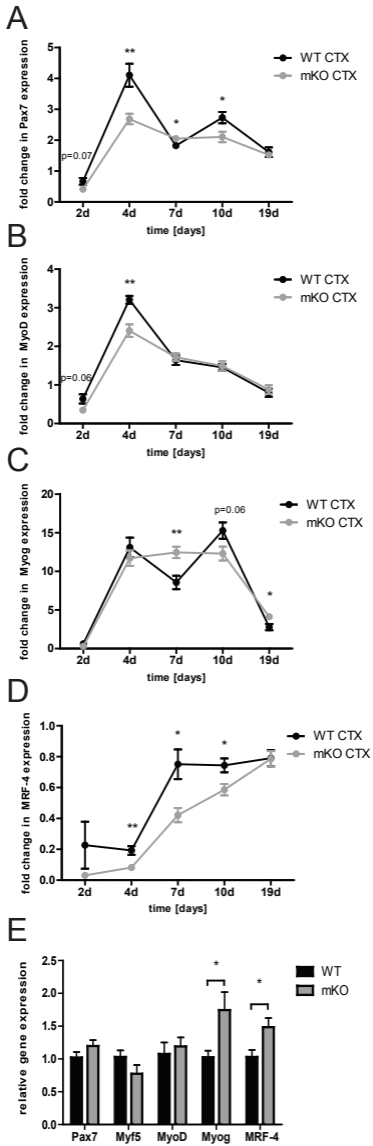

Supplement: Additional file 4: — M1 and M2 marker expression in mKO mice before and in early days after cardiotoxin injury. Relative gene expression A) in the basal state in mKO mice (n = 4–6 per group), B) 2 days (n = 4 per group), and C) 4 days (n = 5 per group) after CTX in mKO mice; CTX-injured muscle gene expression levels were normalized to PBS levels of WT animals; values are plotted as average ± SEM; *p ≤ 0.05, **p ≤ 0.01. (PDF 525 kb) [file 13395_2016_110_MOESM4_ESM.pdf]

A

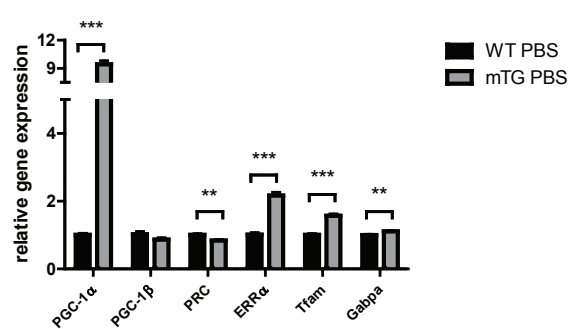

B

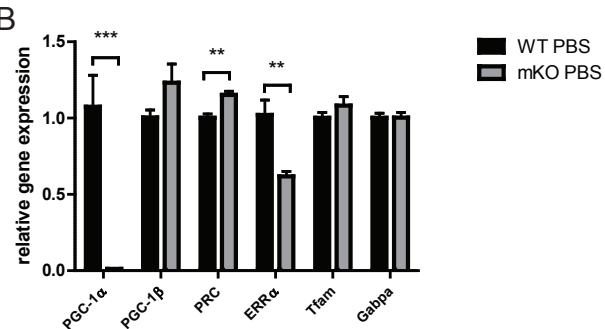

C

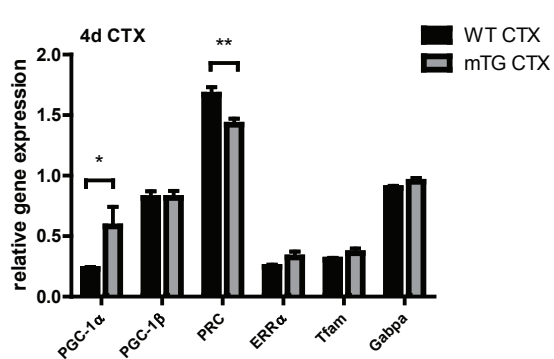

D

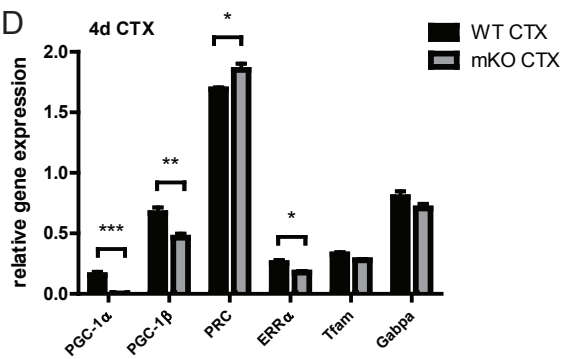

E

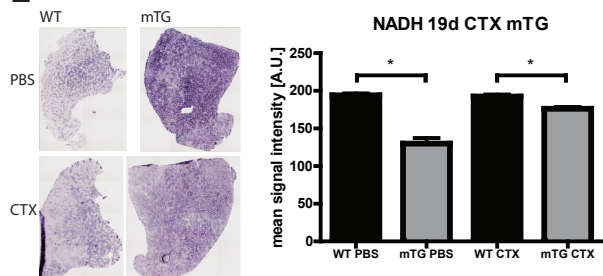

F

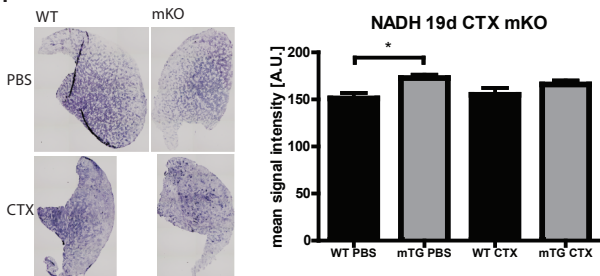

Supplement: Additional file 5: — Regeneration in mKO mice. A) Pax7, B) MyoD, C) Myog, and D) MRF-4 gene expression 2 days (n = 4 per group), 4 days (n = 5 per group), 7 days (n = 5 per group), 10 days (n = 8–12 per group), and 19 days (n = 5 per group) after CTX injury; CTX-injured muscle gene expression levels were normalized to PBS levels of corresponding genotype; E) relative gene expression of the same genes in the basal, uninjured state (n = 6 per group); mKO expression was normalized to WT levels (t test); values are plotted as average ± SEM; *p ≤ 0.05, **p ≤ 0.01. (PDF 535 kb) [file 13395_2016_110_MOESM5_ESM.pdf]

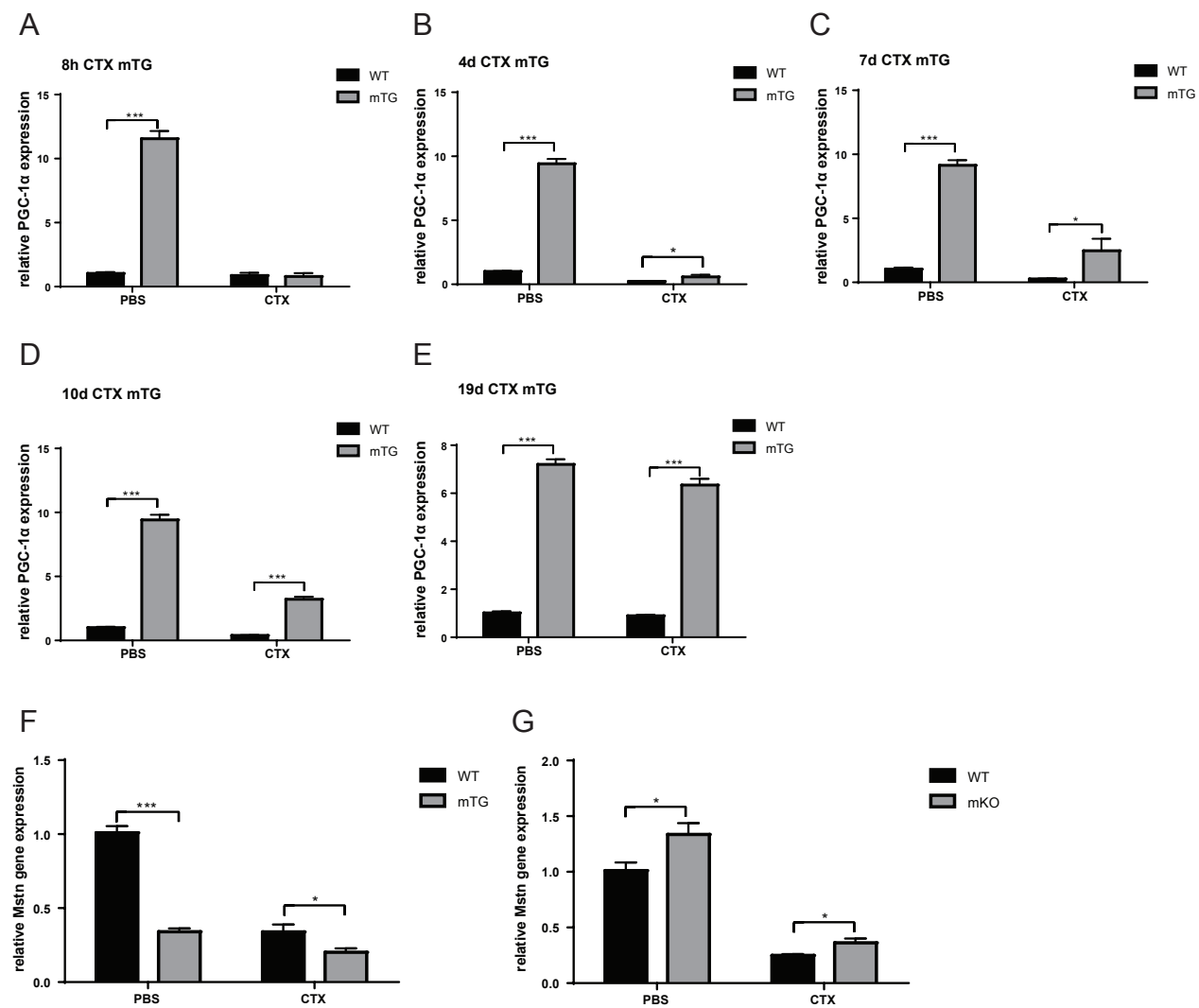

Supplement: Additional file 6: — PGC-1α and Mstn gene expression during course of cardiotoxin-induced regeneration. Relative mRNA levels of PGC-1α in mTG mice A) 8 h (n = 4–6 per group), B) 4 days (7–8 per group), C) 7 days (n = 5 per group), D) 10 days (n = 8 per group), and E) 19 days (n = 8–10 per group) post-CTX; relative mRNA levels of Mstn 19 days after single CTX injection in F) mTG (n = 8–10 per group) and G) mKO (n = 5 per group) mice; values are plotted as average ± SEM; *p ≤ 0.05, ***p ≤ 0.001. (PDF 185 kb) [file 13395_2016_110_MOESM6_ESM.pdf]
